# Perioperative aspirin improves neurological outcome after focal brain ischemia possibly via inhibition of Notch 1 in rat

**DOI:** 10.1186/1742-2094-11-56

**Published:** 2014-03-25

**Authors:** Zhongxing Wang, Wenqi Huang, Zhiyi Zuo

**Affiliations:** 1 Department of Anesthesiology, University of Virginia, 1 Hospital Drive, PO Box 800710, Charlottesville, Virginia 22908-0710, USA; 2 Department of Anesthesiology, First Affiliated Hospital, Sun Yat-Sen University, Guangzhou, China

**Keywords:** Aspirin, Brain ischemia, Inflammatory cytokines, Notch

## Abstract

**Background:**

Perioperative discontinuation of aspirin is often considered due to bleeding concern. We determined whether this discontinuation affected neurological outcome after brain ischemia.

**Methods:**

Adult male Sprague–Dawley rats were subjected to a 90-minute right middle cerebral arterial occlusion (MCAO). They received 30 mg/kg/day aspirin via gastric gavage: 1) for 2 days at 5 days before MCAO; 2) for 2 days at 5 days before MCAO and for 3 days after MCAO; 3) for 7 days before MCAO; or 4) for 7 days before MCAO and for 3 days after MCAO. Neurological outcome was evaluated 3 days after the MCAO. Ischemic penumbral cortex was harvested 1 or 3 days after MCAO for determining Notch intracellular domain (NICD), IL-6 and IL-1β levels.

**Results:**

Aspirin given by regimen 2 and 3 but not by regimen 1 improved neurological outcome. Neuroprotection was achieved by N-[N-(3,5-Difluorophenacetyl)-L-alanyl]-S-phenylglycine t-butyl ester (DAPT), a Notch activation inhibitor. DAPT and aspirin given only by regimen 2 and 3 reduced NICD, IL-6 and IL-1β in the ischemic penumbral cortex. NICD was found in microglial nuclei. Microglial activation in the ischemic tissues was inhibited by aspirin.

**Conclusion:**

Aspirin use during the perioperative period provides neuroprotection. Inhibition of Notch activation and neuroinflammation may contribute to the neuroprotection of aspirin.

## Introduction

Aspirin is commonly used in patients who have risks for cardiovascular events [1]. It can reduce cardiovascular events through multiple mechanisms including anti-platelet actions [2,3]. Studies have shown that giving aspirin before or repeated doses after brain ischemia reduces brain infarct volume and neurological deficits [4,5]. However, aspirin may be discontinued during the perioperative period due to the concern of bleeding in the wound [6,7]. It is not clear whether this discontinuation affects the neurological outcome if an episode of brain ischemia occurs, which is not an infrequent event during the perioperative period.

The mechanisms for aspirin to improve neurological outcome after brain ischemia are not clear. One possible mechanism is to reduce neuroinflammation that is a major secondary insult to cause cell injury after brain ischemia [8]. Aspirin has been shown to reduce brain ischemia-induced neuroinflammation [9]. However, it is not clear how aspirin can reduce neuroinflammation. Recently, it has been shown that brain ischemia can activate Notch, which then contributes to induce neuroinflammation [10].

Based on the above information, we hypothesize that discontinuation of aspirin use during the perioperative period abolishes the neuroprotective effects of aspirin and that the aspirin-induced neuroprotection is mediated by reducing Notch activation and the subsequent neuroinflammation. To address these hypotheses, we simulated different clinical scenarios of perioperative aspirin use and subjected rats to a transient focal brain ischemia. The Notch 1 activation was assessed by measuring the level of Notch intracellular domain (NICD).

## Materials and methods

The animal protocol was approved by the Institutional Animal Care and Use Committee of the University of Virginia (Charlottesville, VA, USA). All animal experiments were carried out in accordance with the National Institutes of Health Guide for the Care and Use of Laboratory Animals (National Institutes of Health publications number 80–23, revised in 1996). Efforts were made to minimize the number used and suffering of animals. Our manuscript was written up in accordance with the Animal Research: Reporting *In Vivo* Experiments.

### Experimental protocols

**Figure 1:**
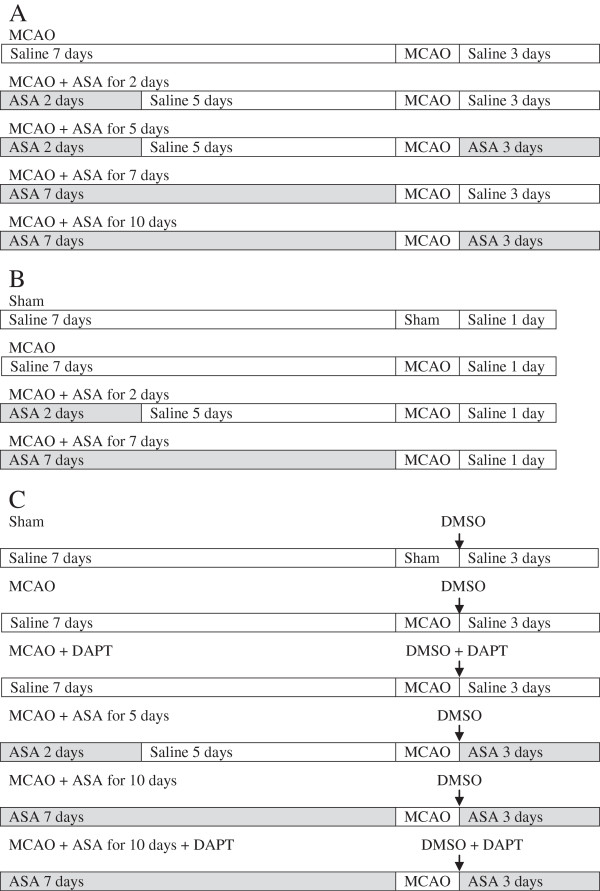
**Diagrams of experimental protocols. (A)** Protocol for experiment 1. **(B)** Protocol for experiment 2. **(C)** Protocol for experiments 3 and 4. ASA, acetylsalicylic acid ; DAPT, N-[N-(3,5-Difluorophenacetyl)-L-alanyl]-S-phenylglycine t-butyl ester; DMSO, dimethyl sulfoxide; MCAO, middle cerebral arterial occlusion.

Four experiments were performed. In experiment 1, male Sprague–Dawley rats weighing 270 to 300 g (Charles River Laboratories Inc., Wilmington, MA, USA) were randomly assigned to receive aspirin (Sigma Aldrich, Saint Louis, MO, USA) via gastric gavage at a dose of 30 mg/kg/day (Figure 1): 1) for 2 days at 5 days before a 90-minute right middle cerebral artery occlusion (MCAO); 2) for 2 days at 5 days before the MCAO and then for 3 days starting from the surgery day to create the MCAO; 3) for 7 days before the MCAO; and 4) for 7 days before the MCAO and for 3 days starting from the surgery day to create the MCAO. An additional group was included in the randomization. This group of rats received saline via gastric gavage for 10 days during the peri-MCAO period. The neurological outcome was evaluated 3 days after the MCAO (n = 10 per group).

Experiment 2 was performed in a similar way as for experiment 1 but without the second and the fourth aspirin regimen. A sham operated group was included in this experiment (Figure 1). The right frontal cortex area 1 (Fr1) was harvested at 24 hours after the MCAO to measure NICD by western blot and the levels of IL-6 and IL-1β by ELISA (n = 8).

In experiment 3, N-[N-(3,5-Difluorophenacetyl)-L-alanyl]-S-phenylglycine t-butyl ester (DAPT; Sigma Aldrich), a Notch activation inhibitor, was used. Rats were randomly divided into five groups (Figure 1). This included the saline group and the groups with aspirin regimen 2 and 4 as described for experiment 1. In the DAPT group, 0.03 mg/kg DAPT was injected into right cerebral ventricle immediately after the 90-minute MCAO. The fifth group was the combination of DAPT therapy and the aspirin treatment of regimen 4. Neurological outcome was evaluated at 3 days after the MCAO (n = 6).

Experiment 4 was performed in the same way as for experiment 3. In addition, a sham operated group was included (Figure 1). The right Fr1 was harvested at 3 days after MCAO for measuring the expression of NICD, IL-6 and IL-1β (n = 8).

### Drug application

Aspirin solution (6 mg/ml) was prepared freshly each day by dissolving the powder in normal saline. Application of 1.5 ml aspirin solution or saline was performed to rats in the morning via gastric gavage (Fine Science Tools, Foster City, CA, USA).

DAPT solution (1 μg/μl) was prepared by dissolving DAPT powder in 0.01 M phosphate-buffered saline containing 5% dimethyl sulfoxide (Fisher Scientific, Fair Lawn, NJ, USA). The solution was filtered and stereotactically injected into the right cerebral ventricle using the following coordinates: -0.8 mm anteroposterior, ±1.5 mm mediolateral, and -4.5 mm dorsoventral from the bregma [11]. The rats that did not require DAPT injection in experiments 3 and 4 received injection of the vehicle for DAPT in the same way.

### Transient middle cerebral arterial occlusion

Right MCAO was created as we have described before [12]. Briefly, rats were induced with isoflurane, intubated and mechanically ventilated with 2% isoflurane. A temperature probe was placed in the temporalis muscle. A servo-controlled warming blanket was used to maintain the temporalis muscle temperature at 37°C. The MCAO was achieved by advancing a 3–0 monofilament nylon suture (Beijing Sunbio Biotech Co. Ltd, Beijing, China) with a rounded tip to the right internal carotid artery via the external carotid artery until slight resistance was felt. Rat’s heart rate and pulse oximeter oxygen saturation were monitored continuously and noninvasively using a MouseOX Murine Plus Oximeter System (Starr Life Sciences Corporation, Oakmont, PA, USA). Isoflurane anesthesia was stopped once the suture was in place. Rats were re-anesthetized by isoflurane at 90 minutes after the onset of MCAO to remove the suture. All animals with surgery received infiltration to the surgical wound with 0.25% bupivacaine before general anesthesia was stopped.

### Evaluation of motor coordination, neurological deficit scores, infarct volumes and hemorrhagic volumes

Motor coordination was evaluated by using an accelerating rotarod as we have described before [13]. Each rat was tested three times in the formal test. The latency and speed of the rat falling off the rotarod were recorded. The speed–latency index (latency in seconds × speed in rpm) of each of the three tests was calculated and averaged for reporting.

Neurological deficit scores were evaluated by a person blinded to the group assignment based on an eight-point scale as we have described before [14,15].

Infarct volumes were measured after staining of the 2-mm thick slices with 2% 2,3,5-triphenyltetrazolium chloride as we have described before [14,15]. The percentage of infarct volumes in the ipsilateral hemisphere volume was calculated to account for cerebral edema and differential shrinkage from brain ischemia and tissue processing and to correct for individual differences in brain volumes. The hemorrhagic volumes were measured in the same way as for measuring infarct volumes, but measurements were performed before the slices were stained by 2,3,5-triphenyltetrazolium chloride because the staining process would wash away blood in the brain slices.

### Brain tissue harvesting

Rats were killed by deep isoflurane anesthesia and transcardially perfused with normal saline at 1 day or 3 days after the MCAO. The Fr1, an ischemic penumbral region in this model [15], between bregma + 2 and 0 mm was harvested for Western analysis of NICD expression and ELISA of IL-6 and IL-1β.

### Western analysis

Fr1 tissues were homogenized in buffer A (10 mM HEPES, 1.5 mM MgCl_2_, 10 mM KCl, 0.5 mM DTT, 0.05% NP40, pH 7.9) with protease inhibitors (10 mg/ml aproteinin, 5 mg/ml peptastin, 5 mg/ml leupeptin, and 1 mM phenylmethanesulfonylfluoride) on ice as we have described before [16]. The homogenates were centrifuged at 4°C at 13,000 rpm for 25 minutes. The supernatant was harvested and used for Western blotting.

The primary antibodies used were the rabbit anti-Notch1 NICD antibody (1:1000; Cell Signaling Technology, Danvers, MA, USA) and the rabbit anti-β-Actin antibody (1:4000; Cell Signaling Technology). A secondary horseradish peroxidase-conjugated goat anti-rabbit antibody (1:5,000; Pierce, Rockford, IL, USA) was used. The protein bands were visualized with the enhanced chemiluminescence methods. Quantitative analysis of the protein bands was performed using an Image-Quant 5.0 GE Healthcare Densitometer (GE Healthcare, Sunnyvale, CA, USA). The densities of NICD protein bands were normalized to those of β-Actin proteins from the same sample to control for errors in protein sample loading and transferring during Western analysis.

### ELISA assay of cytokines in the brain tissues

IL-1β and IL-6 levels in the Fr1 tissues were determined with Quantikine ELISA kits (R&D Systems, Minneapolis, MN, USA) according to the manufacturer’s instructions as we have described before [17,18]. Briefly, brain tissues were homogenized on ice in 20 mMTris–HCl buffer (pH 7.3) containing protease inhibitors (10 mg/ml aproteinin, 5 mg/ml peptastin, 5 mg/ml leupeptin, and 1 mM phenylmethanesulfonylfluoride). Homogenates were centrifuged at 10,000 g for 10 minutes at 4°C. The supernatant was then ultracentrifuged at 150,000 g for 2 hours at 4°C. Bradford protein assay of the supernatant was performed for each sample. The supernatant was used in ELISA. The quantity of IL-1β and IL-6 in each brain sample was standardized to its protein contents.

### Immunofluorescent staining

The staining and quantification of the staining were performed as we have described before [19]. Briefly, rats were killed by deep isoflurane anesthesia and transcardially perfused with 4% paraformaldehyde at 1 day after the MCAO. Brains were harvested, fixed in 4% paraformaldehyde at 4°C for 18 hours and then embedded in paraffin. Coronal sections at 5 μm were mounted on slides. Antigen retrieval was performed in sodium citrate buffer (10 mM sodium citrate, 0.05% Tween 20, pH 6.0) at 95 to 100°C for 20 minutes. The sections were then incubated with 5% normal donkey serum and 1% bovine serum albumin in Tris-buffered saline for 2 hours at room temperature.

To stain ionized calcium binding adapter molecule 1 (Iba-1) for its quantification, the sections were incubated at 4°C overnight with rabbit polyclonal anti-Iba-1 antibody (1:500; Wako Chemicals USA, Richmond, VA, USA) and then rinsed in Tris-buffered saline containing 0.025% triton-X 100. The donkey anti-rabbit IgG antibody conjugated with Alexa Fluor 488 (1:200; Invitrogen, Eugene, ON, USA) was applied for 1 hour at room temperature in a dark room. To stain Iba-1 for its co-localization with NICD, sections were incubated with goat polyclonal anti-Iba-1 antibody (1:200; Abcam, Cambridge, MA, USA) and then donkey anti-goat IgG antibody conjugated with Alexa Fluor 488 (1:200; Invitrogen). For glial fibrillary acidic protein (GFAP) staining, the mouse monoclonal anti-GFAP (1:300; Chemicon, Temecula, CA, USA) and the donkey anti-mouse IgG antibody conjugated with NL493 (1:200; R&D Systems, Minneapolis, MN, USA) were applied in the same way as for staining Iba-1. The antibodies used to stain microtubule-associated protein 2 (MAP-2) were mouse monoclonal anti-MAP-2 antibody (1:300; Abcam) and the donkey anti-mouse IgG antibody conjugated with NL493. To stain NICD, the rabbit anti-Notch1 NICD antibody (1:100; R&D Systems) and the donkey anti-rabbit IgG antibody conjugated with NL557 (1:200; R&D Systems) were applied. Images were acquired with a fluorescence microscope with a charge-coupled device camera. A negative control omitting the incubation with the primary antibody was included in all experiments.

For quantification of Iba-1 staining, three independent microscopic fields in each section were randomly acquired in the Fr1 area, and three sections per rat were imaged. The number of pixels per image with intensity above a predetermined threshold level was considered to be positively stained areas. This measurement was performed by using the Image J 1.47n software. The degree of positive immunoreactivity was reflected by the percentage of the positively stained area in the total area of the image. All quantitative analyses were performed in a blinded fashion.

### Statistical analysis

Parametrical data are presented as means ± SEM. The results of speed–latency index ratio, infarct volume, edema index, western blotting, ELISA and Iba-1 immunofluorescent staining were analyzed by one-way analysis of variance, followed by the Tukey test. Neurologic deficit scores were analyzed by one-way analysis of variance on ranks, followed by the Tukey test. A *P* < 0.05 was accepted as significant. All statistical analyses were performed using the SigmaStat program (SYSTAT Software Inc., Point Richmond, CA, USA).

## Results

**Figure 2:**
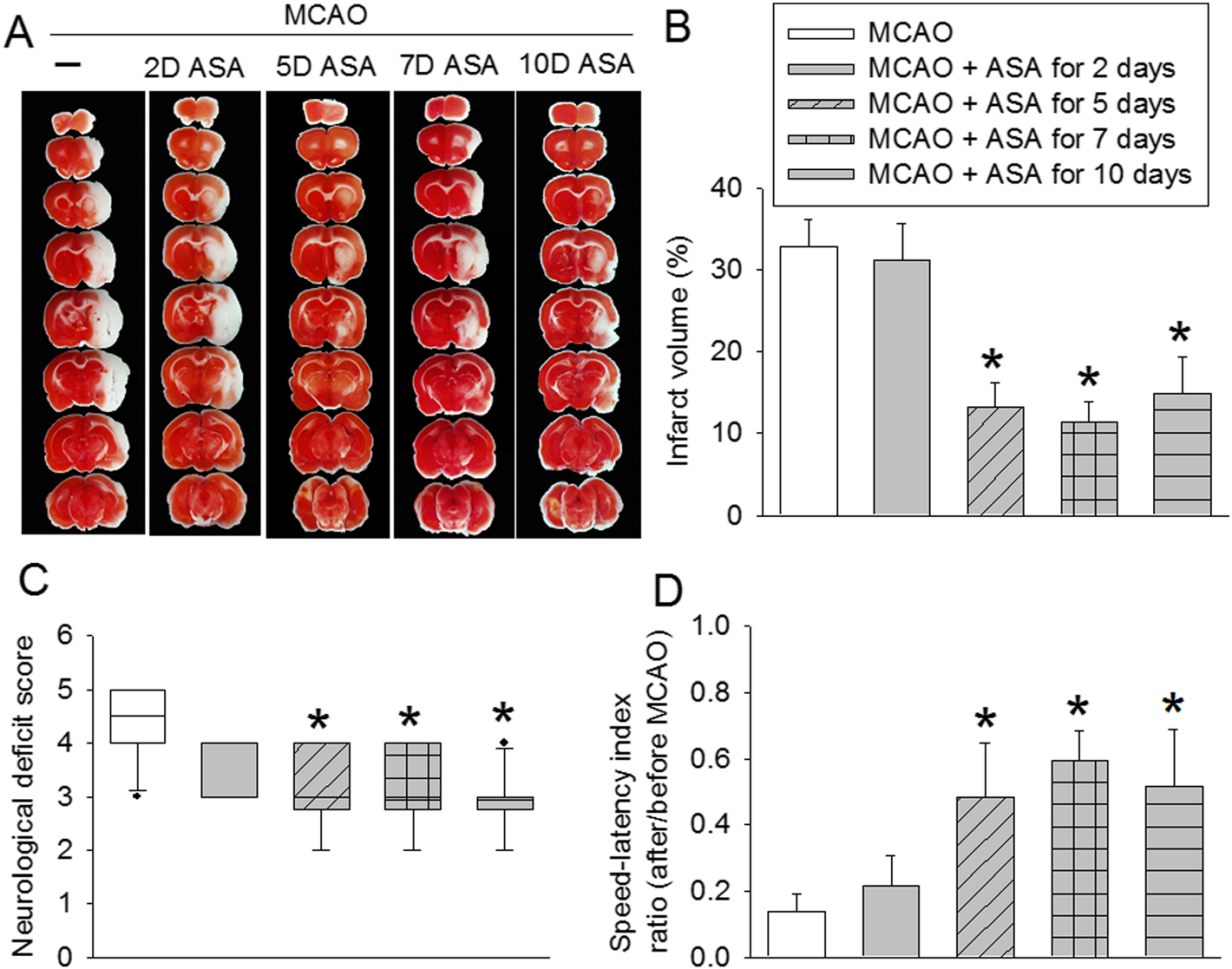
**Aspirin-induced neuroprotection.** Rats received various aspirin treatments around a 90-minute middle cerebral arterial occlusion (MCAO). The results were evaluated at 3 days after the MCAO. **(A)** Brain slices stained with 2,3,5-triphenyltetrazolium chloride from representative mice. **(B)** Percentage of brain infarct volume in ipsilateral hemisphere volume. **(C)** Neurological deficit scores evaluated immediately before the animals were euthanized for the assessment of infarct sizes (data are presented in panel B). ●, Lowest or highest score (the score will not show up if it falls in the 95% interval); between lines, 95% interval of the data; inside boxes, 25-75% interval including the median of the data. **(D)** The performance on rotarod. Rats were tested before and 3 days after the MCAO and the speed–latency index ratio of these two tests are presented. All results except for those in panel C are the means ± SEM (n = 10). *P < 0.05, compared with the animals subjected to MCAO only. ASA, acetylsalicylic acid.

No animals had an episode of hypoxia (defined as oxygen saturation of arterial blood <90%) during the surgery to create MCAO. Use of aspirin for 2 days at 5 days before the brain ischemia did not affect the brain infarct volumes and neurological functions. However, aspirin use for 2 days at 5 days before the brain ischemia and then for 3 days starting from surgery day to induce the brain ischemia, use of aspirin for 7 days only before the brain ischemia or continuation of aspirin use throughout the perioperative period significantly reduced brain infarct volumes and improved neurological functions as measured by rotarod performance and neurological deficit scores (Figure 2).

**Figure 3:**
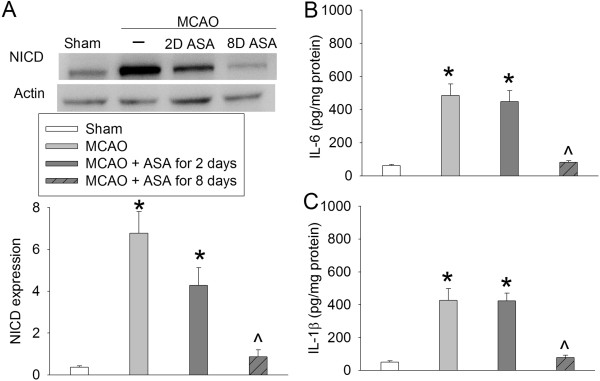
**Aspirin-induced inhibition of Notch activation and proinflammatory cytokine production.** Rats received various aspirin treatments around a 90-minute middle cerebral arterial occlusion (MCAO). The right frontal cortex area 1 was harvested at 24 hours after the MCAO. The cytosol was prepared for Western blotting for Notch intracellular domain (NICD) and ELISA for IL-6 and IL-1β. **(A)** NICD expression. Top panel shows representative Western blots and bottom panel shows the quantification results. **(B)** IL-6 results. **(C)** IL-1β results. Results are the means ± SEM (n = 8). *P < 0.05, compared with sham operated animals; ^*P* < 0.05, compared with the animals subjected to MCAO only. ASA, acetylsalicylic acid.

**Figure 4:**
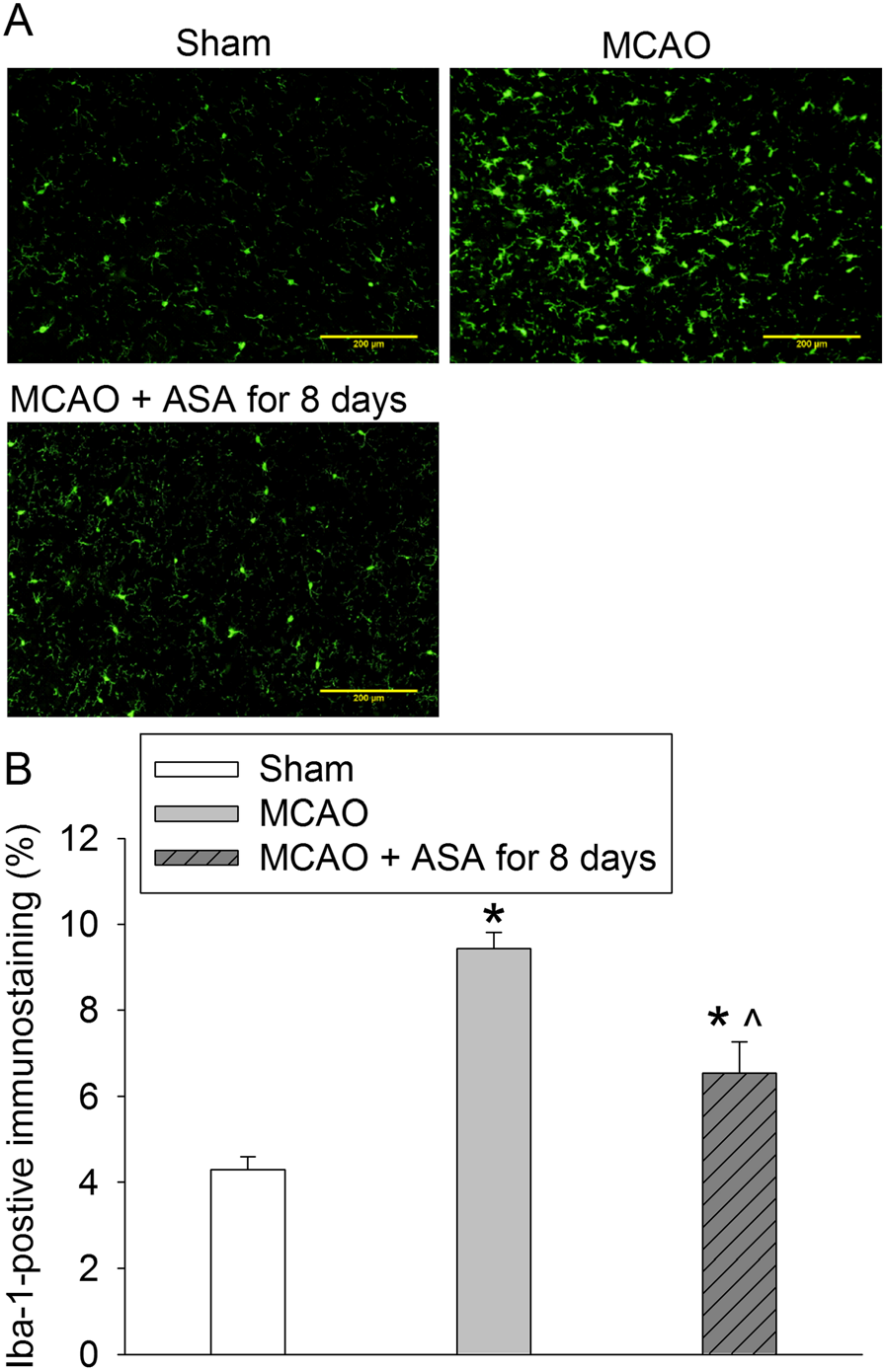
**Aspirin attenuated ionized calcium binding adapter molecule 1** (**Iba-1) expression in the ischemic penumbral tissues.** Rats received or did not receive aspirin for 7 days before a 90-minute middle cerebral arterial occlusion (MCAO). Brain was harvested 24 hours after the MCAO for immunostaining of Iba-1. **(A)** Representative immunostaining images. Scale bars = 200 μm. **(B)** Graphic presentation of the percentage area that is Iba-1-positive stained in the total area of the image. Values presented as mean ± SEM (n = 8). *P < 0.05, compared with sham; ^*P* < 0.05, compared with MCAO. ASA, acetylsalicylic acid.

**Figure 5:**
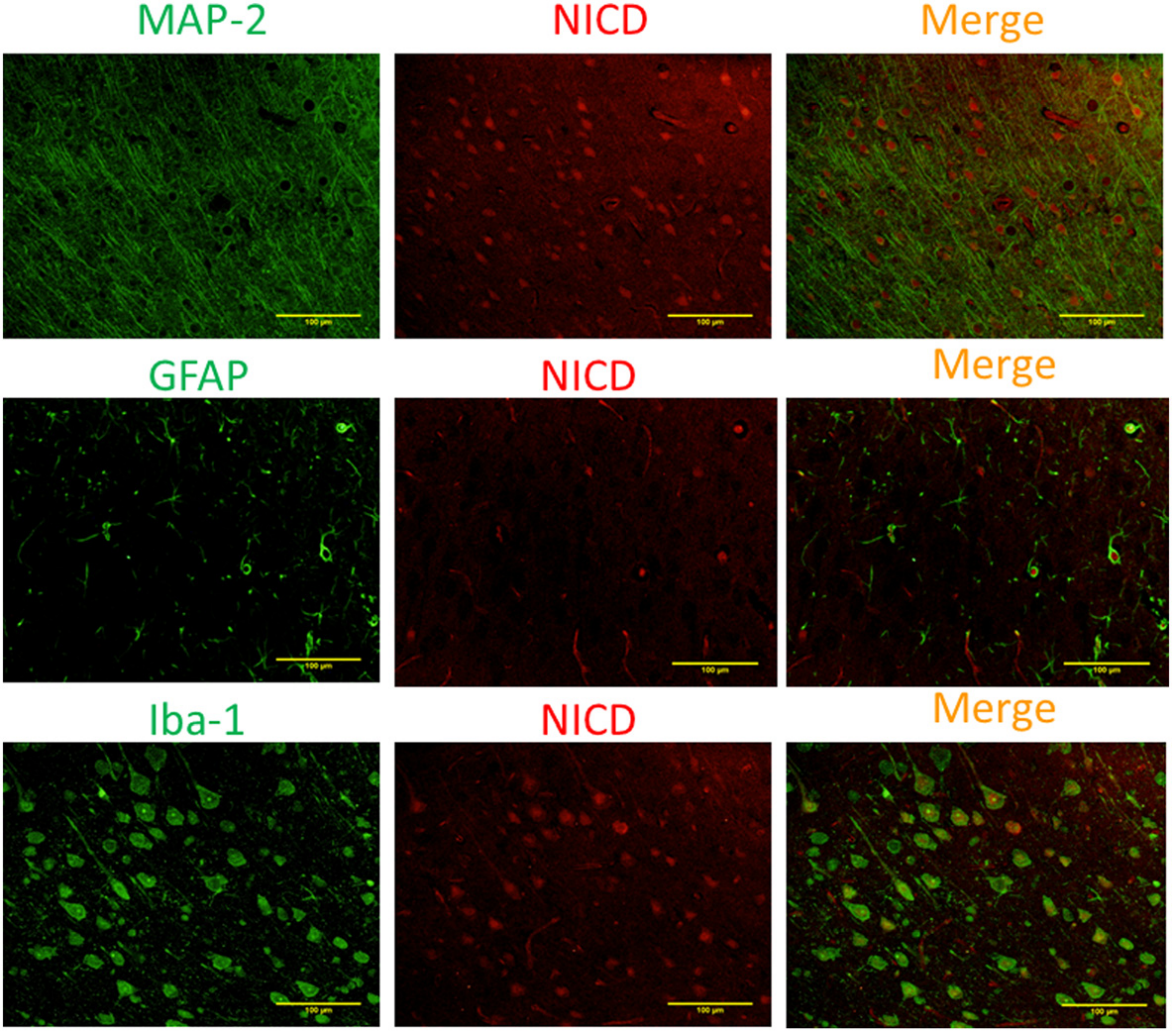
**Cellular localization of Notch intracellular domain (NICD) in the ischemic penumbral brain tissues.** Brain was harvested at 24 hours after a 90-minute middle cerebral arterial occlusion. Representative immunostaining images of frontal cortex area 1 are presented. Scale bars = 100 μm. GFAP, glial fibrillary acidic protein; MAP-2, microtubule-associated protein 2.

There was a significant increase of NICD, IL-6 and IL-1β in the ischemic penumbral tissues. These increases were inhibited by continuous use of aspirin during the perioperative period and were not affected by aspirin use for 2 days at 5 days before the brain ischemia (Figure 3). Similarly, continuous use of aspirin during the perioperative period inhibited the increase of Iba-1, a microglial marker, in the ischemic penumbra (Figure 4). Interestingly, NICD positive staining in the ischemic penumbra appeared in the nuclei of cells that were also positively stained for MAP-2, Iba-1 and GFAP (Figure 5). MAP-2 and GFAP are markers for neurons and astrocytes, respectively.

**Figure 6:**
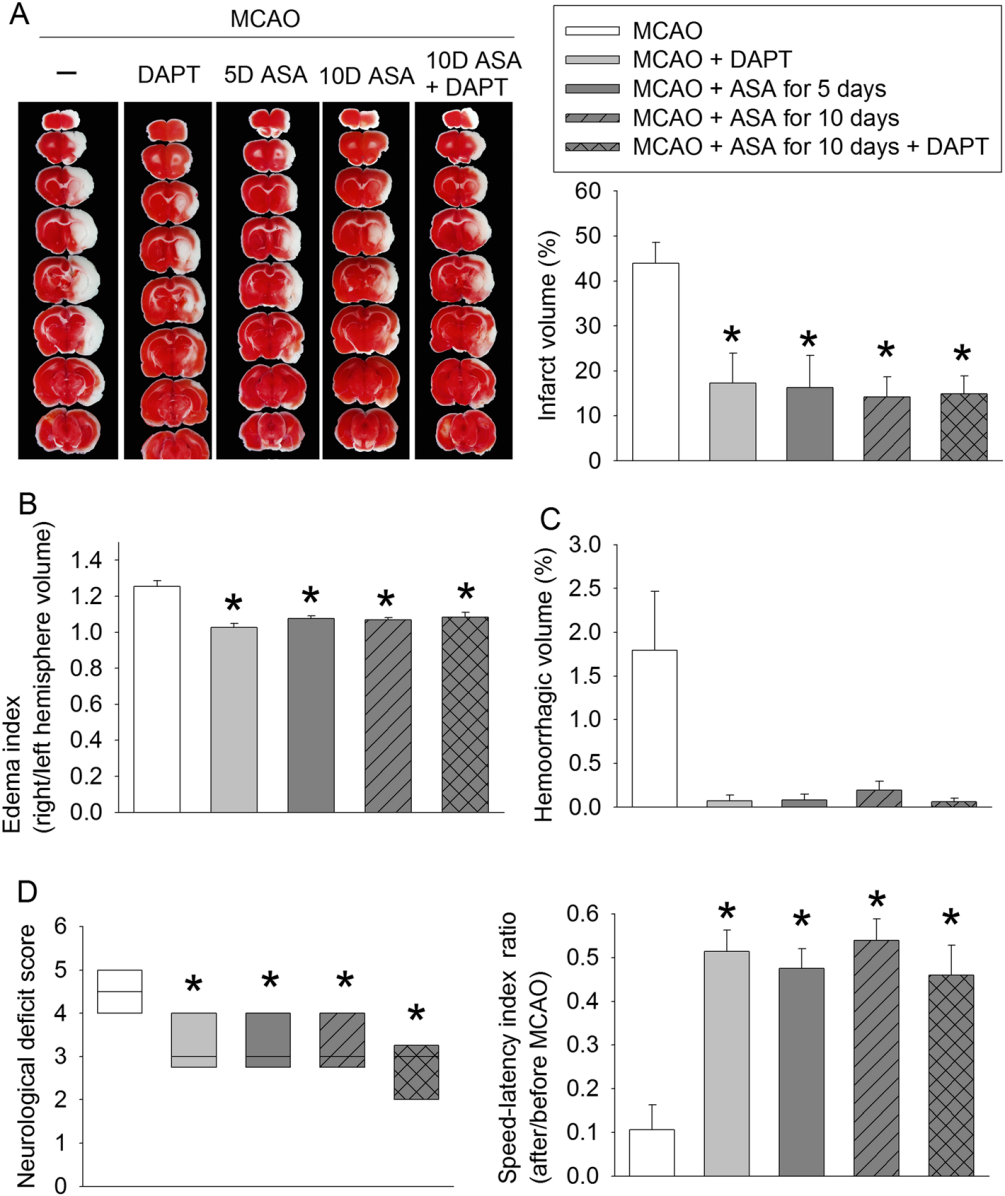
**Aspirin and DAPT-induced neuroprotection.** Rats received various aspirin treatments around or immediately after a 90-minute middle cerebral arterial occlusion (MCAO). Intracerebroventricular injection of N-[N-(3,5-Difluorophenacetyl)-L-alanyl]-S-phenylglycine t-butyl ester (DAPT) was performed immediately after the MCAO. The results were evaluated at 3 days after the MCAO. **(A)** Brain slices stained with 2,3,5-triphenyltetrazolium chloride from representative mice and percentage of brain infarct volume in ipsilateral hemisphere volume. **(B)** Edema index. **(C)** Percentage of hemorrhagic volume in ipsilateral hemisphere volume. **(D)** Left panel shows the neurological deficit scores evaluated immediately before the animals were euthanized for the assessment of infarct sizes (data are presented in panel A). ●, Lowest or highest score (the score will not show up if it falls in the 95% interval); between lines, 95% interval of the data; inside boxes, 25-75% interval including the median of the data. Right panel shows the performance on rotarod. Rats were tested before and 3 days after the MCAO and the speed–latency index ratio of these two tests are presented. All results except for those in the left panel of panel D are the means ± SEM (n = 6). *P < 0.05, compared with the animals subjected to MCAO only. ASA, acetylsalicylic acid.

Application of DAPT, a Notch activation inhibitor, immediately after brain ischemia reduced brain infarct volumes and edema as well as improved neurological functions. Similar results were observed with aspirin use for 2 days at 5 days before the brain ischemia and then for 3 days starting from surgery day to induce the brain ischemia, continuation of aspirin use throughout the perioperative period, and the combination of DAPT and aspirin use throughout the perioperative period. These uses of aspirin and DAPT had a trend of reducing hemorrhagic volumes but none of these reductions reached statistical significance (Figure 6).

**Figure 7:**
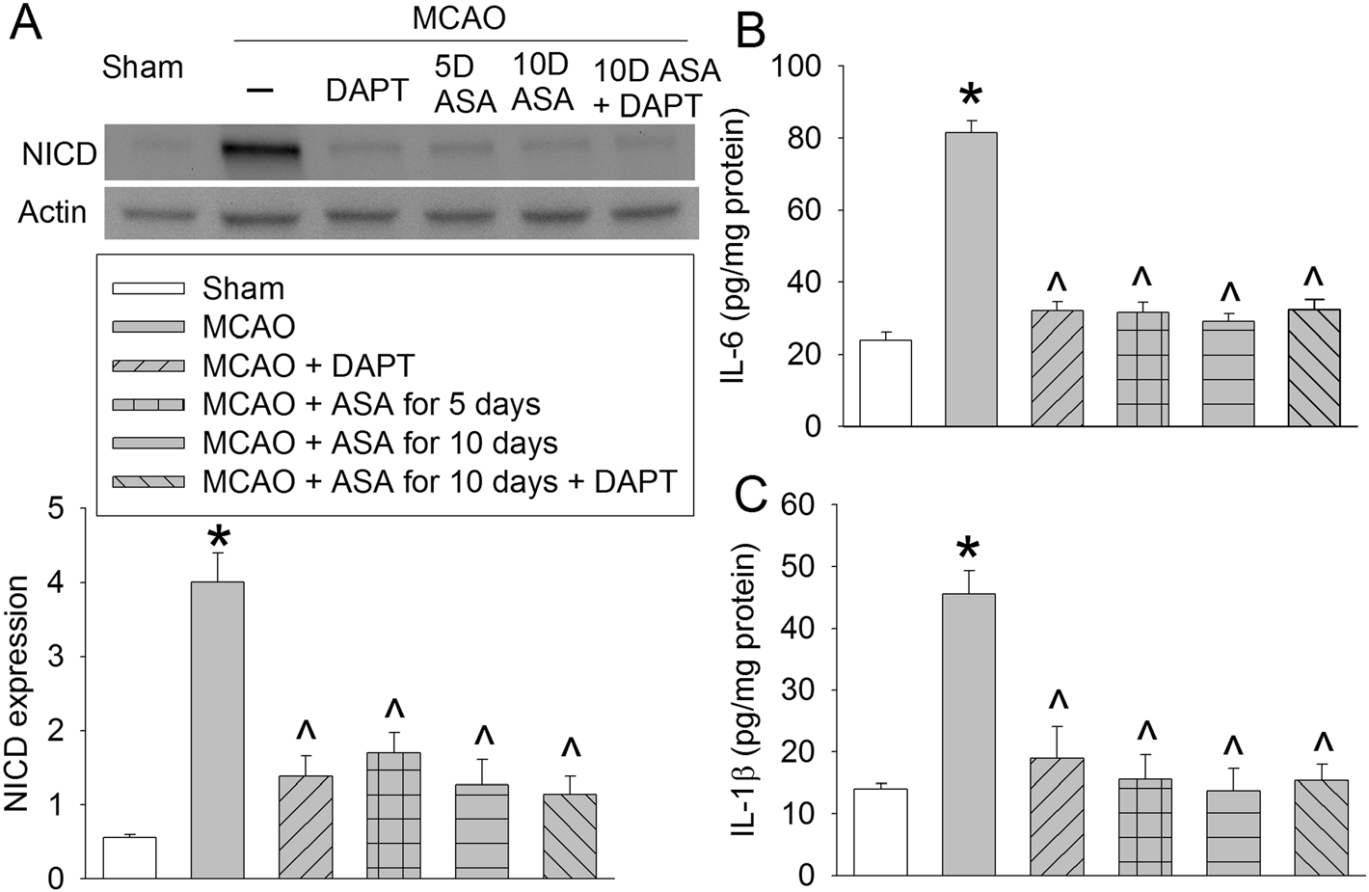
**Aspirin-induced inhibition of Notch activation and proinflammatory cytokine production.** Rats received various aspirin treatments around a 90-minute middle cerebral arterial occlusion (MCAO). Intracerebroventricular injection of N-[N-(3,5-Difluorophenacetyl)-L-alanyl]-S-phenylglycine t-butyl ester (DAPT) was performed immediately after the MCAO. The right frontal cortex area 1 was harvested at 3 days after the MCAO. The cytosol was prepared for Western blotting for Notch intracellular domain (NICD) and ELISA for IL-6 and IL-1β. **(A)** NICD expression. Top panel shows representative Western blots and bottom panel shows the quantification results. **(B)** IL-6 results. **(C)** IL-1β results. Results are the means ± SEM (n = 8). *P < 0.05, compared with sham; ^*P* < 0.05, compared with the animals subjected to MCAO only. ASA, acetylsalicylic acid.

As expected, DAPT significantly reduced the expression of NICD, IL-6 and IL-1β in the ischemic penumbral brain tissues. Similarly, aspirin use for 2 days at 5 days before the brain ischemia and then for 3 days starting from surgery day to induce the brain ischemia, continuation of aspirin use throughout the perioperative period, and the combination of DAPT and aspirin use throughout the perioperative period also reduced the expression of NICD, IL-6 and IL-1β in the ischemic penumbral brain tissues (Figure 7).

## Discussion

Perioperative stroke rate ranges from 0.2% in patients after total joint arthroplasty [20] to 3.3% in patients after carotid endarterectomy [21]. Although the preventive effects of aspirin on cardiovascular events are well documented [1], discontinuation of aspirin use during the perioperative period is often considered due to the concern of bleeding [6]. In fact, the new guidelines from the American College of Chest Physicians include discontinuation of aspirin use for 7 to 10 days before the surgery for patients with low risks for cardiovascular events [7]. However, it is not clear whether discontinuation of aspirin during the perioperative period affects the neurological outcome if brain ischemia occurs. Our results clearly showed improved neurological outcome by continuation of the aspirin regimen. An alternative approach to achieve this beneficial effect is to start aspirin therapy immediately after brain ischemia if aspirin regimen has to be stopped prior to surgery because our study showed that aspirin therapy for 2 days at 5 days before the MCAO and then for 3 days starting from surgery day to create the MCAO also improved the neurological outcome. Similarly, aspirin use only before the brain ischemia for 7 days also provided significant protection, suggesting that post-brain ischemia aspirin use may not be critical for the protection.

Neuroinflammation is a major secondary insult to cause cell injury and death after brain ischemia [8]. A recent study suggests that Notch activation plays an important role in neuroinflammation after brain ischemia [10]. When Notch in the plasma membrane is bound with its ligands presented by a neighboring cell, Notch is activated. This activation produces NICD that can travel to nuclei to induce expression of various genes including proinflammatory cytokines [22]. Our studies showed that NICD was increased in the ischemic penumbral tissues and that this increase was abolished by DAPT. DAPT also inhibited the increased IL-6 and IL-1β in the ischemic tissues and improved neurological outcome. These results suggest that brain ischemia-induced Notch activation and the subsequent proinflammatory cytokines contribute to ischemic brain injury. Consistent with this finding, our previous study showed a critical role of IL-1β in ischemic brain injury [23].

Our studies also showed that aspirin reduced NICD, IL-6 and IL-1β in the ischemic brain tissues. In addition, the combination of aspirin and DAPT did not provide better neuroprotection than DAPT or aspirin alone. These results suggest that inhibition of Notch activation in the ischemic penumbral brain tissues contributes to the neuroprotection of aspirin. In supporting this suggestion, microglial activation as reflected by increased Iba-1 expression in the ischemic brain tissues was inhibited by aspirin use. Microglial activation in the ischemic brain tissues is known to be mediated by Notch signaling [10]. Consistent with this knowledge, our study showed that NICD was expressed in the nuclei of microglial cells in the ischemic brain tissues. However, the Notch signaling in the neurons and astrocytes of these tissues may also be activated because NICD was found in their nuclei as well. These cells participate in inflammatory responses in the brain because they also can produce inflammatory cytokines, such as IL-1β [18].

In addition to anti-inflammatory effects, aspirin is antipyretic. This effect may also contribute to its neuroprotection because brain ischemia often induces pyrexia that can worsen neurological outcome. However, the pyrexia caused by brain ischemia in rats is resistant to aspirin [24,25]. Thus, the antipyretic effects may not be a mechanism for the neuroprotection induced by aspirin in our study.

Multiple effects in addition to the anti-inflammatory effects may contribute to the neuroprotection of aspirin. For example, aspirin has been shown to preserve ATP levels and reduce extracellular glutamate levels in the ischemic brain tissues [9,26]. This effect can attenuate glutamate excitotoxicity, a major secondary insult leading to cell injury after brain ischemia [8]. Also, peripheral immune responses to stroke can affect the degree of ischemic brain injury [27]. We chose systemic aspirin use to simulate the clinical situation. This use may affect the peripheral immune responses to provide neuroprotection that was observed in this study.

Aspirin use may cause significant bleeding in the surgical wound [6] or intracranial tissues [28,29]. However, the hemorrhagic volumes in the brain tissues were not affected by aspirin use in our study. Future studies are needed to determine whether aspirin increases hemorrhagic transformation in the ischemic brain tissues.

Therapeutic dosages of aspirin in humans for relieving pain and fever are 325 to 650 mg orally or rectally every 4 hours as needed. The dosages for similar purposes in rats are 100 to 150 mg/kg orally every 4 hours as needed [30,31]. Prophylactic doses of aspirin are 75 to 325 mg orally once every day in humans. We used 30 mg/kg aspirin orally once a day in rats in this study, which should fall into the prophylactic dosage window in rats.

In summary, we have shown that continuation of aspirin use improves neurological outcome after focal brain ischemia. This effect may be mediated by inhibition of Notch activation and the subsequent proinflammatory cytokine production in the ischemic penumbral brain tissues.

## Abbreviations

DAPT: N-[N-(3,5-Difluorophenacetyl)-L-alanyl]-S-phenylglycine t-butyl ester; ELISA: enzyme-linked immunosorbent assay; Fr1: right frontal cortex area 1; GFAP: glial fibrillary acidic protein; Iba-1: ionized calcium binding adapter molecule 1; IL: interleukin; MAP-2: microtubule-associated protein 2; MCAO: middle cerebral arterial occlusion; NICD: Notch intracellular domain.

## Competing interests

The authors declare that they have no competing interests.

## Authors’ contributions

ZW and ZZ conceived the study. ZW, WH and ZZ designed the experiments. ZW performed the experiments. ZW and ZZ analyzed the data. ZW drafted the Methods section. ZZ wrote the manuscript. All authors read and approved the final manuscript.
